# Corrigendum: Greenland subglacial drainage evolution regulated by weakly connected regions of the bed

**DOI:** 10.1038/ncomms14501

**Published:** 2017-02-07

**Authors:** Matthew J. Hoffman, Lauren C. Andrews, Stephen F. Price, Ginny A. Catania, Thomas A. Neumann, Martin P. Lüthi, Jason Gulley, Claudia Ryser, Robert L. Hawley, Blaine Morriss

Nature Communications 7: Article number: 13903; DOI: 10.1038/ncomms13903 (2016); Published 12 13 2016; Updated 01 20 2017

The original version of this Article contained a typographical error in the spelling of the author Stephen F. Price, which was incorrectly given as Stephen A. Price. This has now been corrected in both the PDF and HTML versions of the Article.

