# Supplementary material for: Greenland subglacial drainage evolution regulated by weakly connected regions of the bed
Source: Nat Commun. 2016 Dec 19;7:13903. doi: 10.1038/ncomms13903 (PMC5187425; doi:10.1038/ncomms13903)
Supplement: Supplementary Information — Supplementary Figures, Supplementary Tables, Supplementary Methods and Supplementary References. [file ncomms13903-s1.pdf]

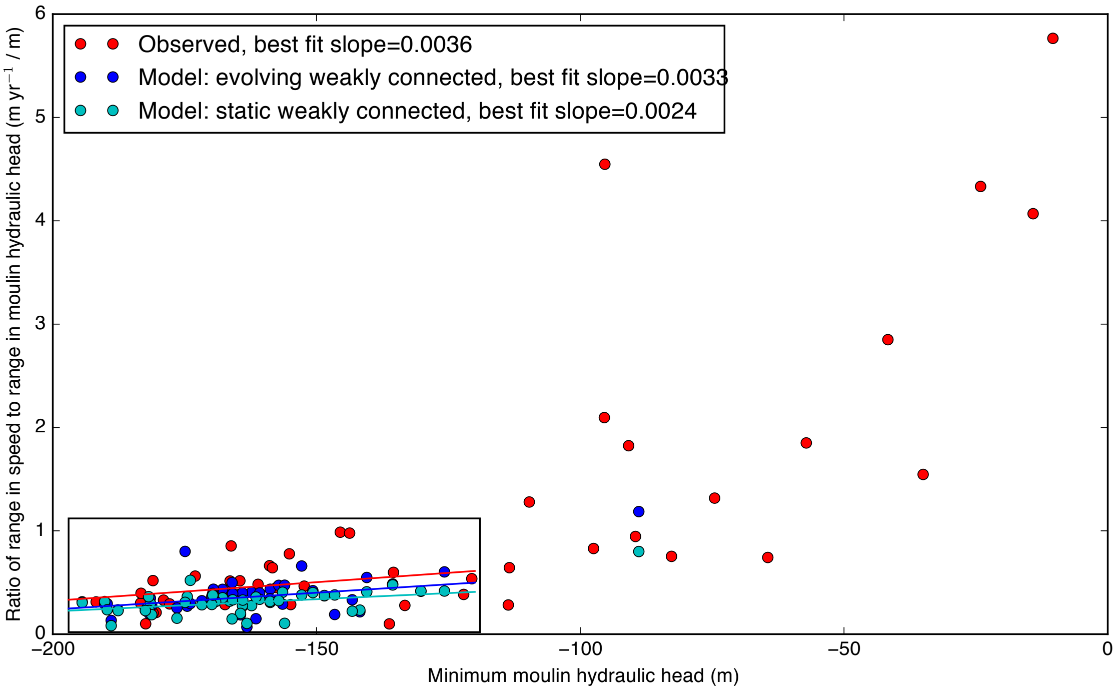

Supplementary Figure 1: **Assessing sensitivity of basal friction law to effective pressure.** Comparison of minimum moulin hydraulic head with the ratio of range in speed to range in moulin hydraulic head on each day. This provides a measure of how the sensitivity of the basal friction law to effective pressure decreases at large effective pressures. See Methods: Ice velocity calculations for detailed description. Each point represents one day and corresponds to a line segment in Fig. 3: the x-axis here is the x-value of the left endpoint of each line segment in Fig. 3, and the y-axis is the slope. Red points are derived from the observations of moulin head and ice surface speed. Blue points are derived from the modeled channel hydraulic head and ice surface speed from the model with an evolving weakly-connected system, and cyan are for the model with static weakly connected system. To confirm model parameter calibration we calculate a line of best fit for the observations and both models. To apply linear regression, we restrict the regression to the region of smallest moulin hydraulic head values (<-120 m), indicated by the black box, where this relationship is approximately linear.

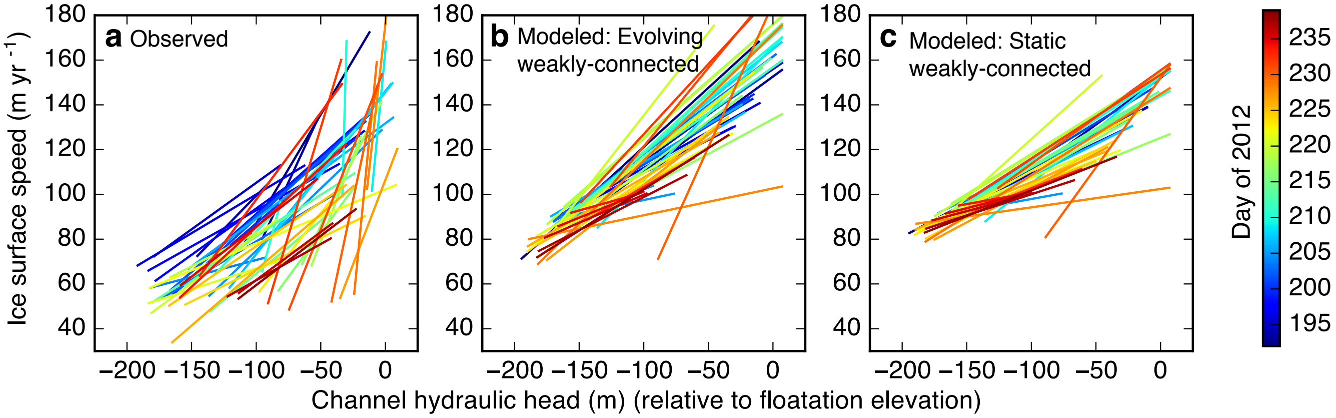

Supplementary Figure 2: **Seasonal evolution of the relationship between subglacial water pressure and ice speed with f_w declining by 0.10.** Each plot shows the minimum and maximum daily values of hydraulic head in the moulin-channel system and ice surface speed for the second half of the 2012 summer. **a** Observed relationship showing seasonal hysteresis of lowering ice speed for the same moulin head as summer progresses. Modified from Andrews et al.[2]. **b** Modeled relationship with f_w declining by 0.10 over the 47 day period plotted rather than changes to the permeability. **c** Modeled relationship from control simulation with static weakly-connected system.

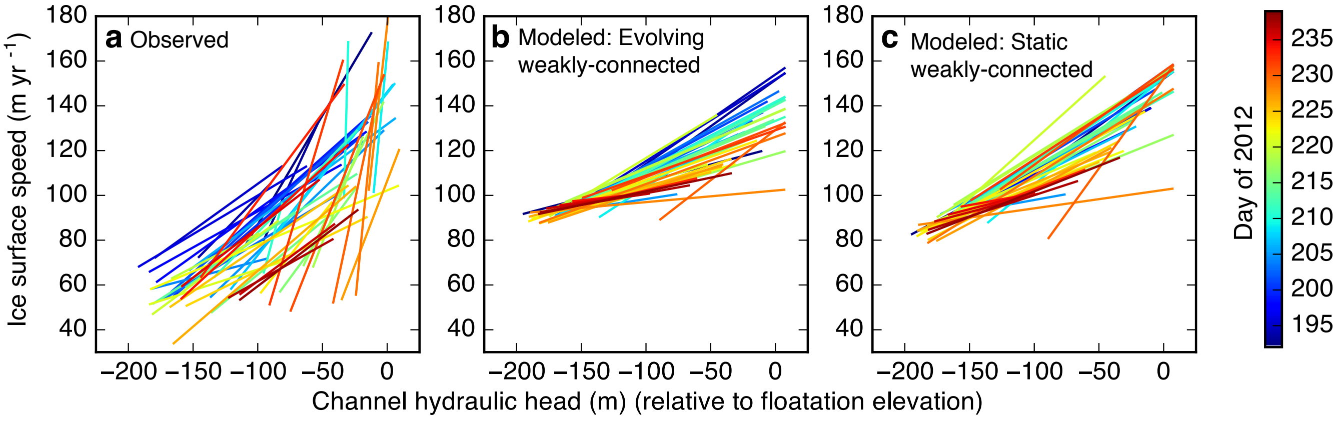

Supplementary Figure 3: **Seasonal evolution of the relationship between subglacial water pressure and ice speed with f_w increasing by 0.10.** Each plot shows the minimum and maximum daily values of hydraulic head in the moulin-channel system and ice surface speed for the second half of the 2012 summer. **a** Observed relationship showing seasonal hysteresis of lowering ice speed for the same moulin head as summer progresses. Modified from Andrews et al.[2]. **b** Modeled relationship with f_w increasing by 0.10 over the 47 day period plotted rather than changes to the permeability. **c** Modeled relationship from control simulation with static weakly-connected system.

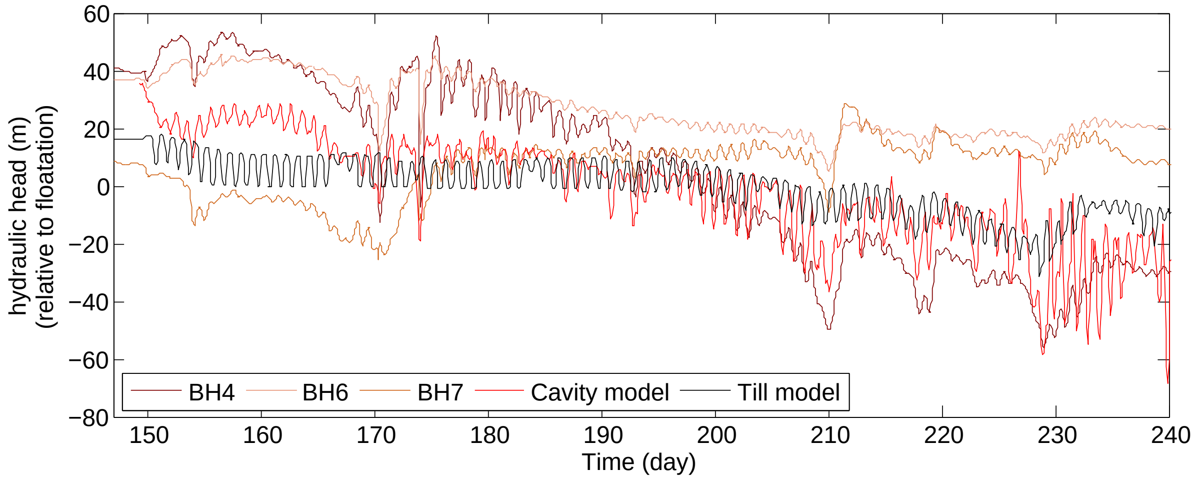

Supplementary Figure 4: **Subglacial till model results.** Hydraulic head in the weakly-connected system as computed by the till model (black) compared to hydraulic head measured in boreholes (pink, orange, and maroon) and the weakly-connected cavity model (red). BH=borehole.

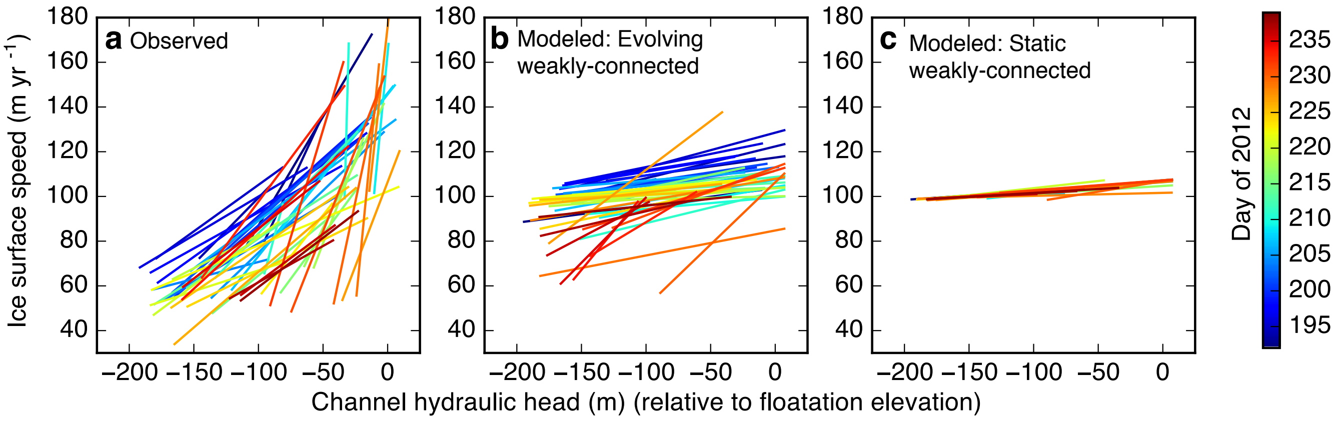

Supplementary Figure 5: **Seasonal evolution of the relationship between subglacial water pressure and ice speed with $f_w = 0.90$.** Each plot shows the minimum and maximum daily values of hydraulic head in the moulin-channel system and ice surface speed for the second half of the 2012 summer. **a** Observed relationship showing seasonal hysteresis of lowering ice speed for the same moulin head as summer progresses. Modified from Andrews et al.[2]. **b** Modeled relationship with increasing permeability of the weakly-connected regions of the bed. **c** Modeled relationship from control simulation with static weakly-connected system.

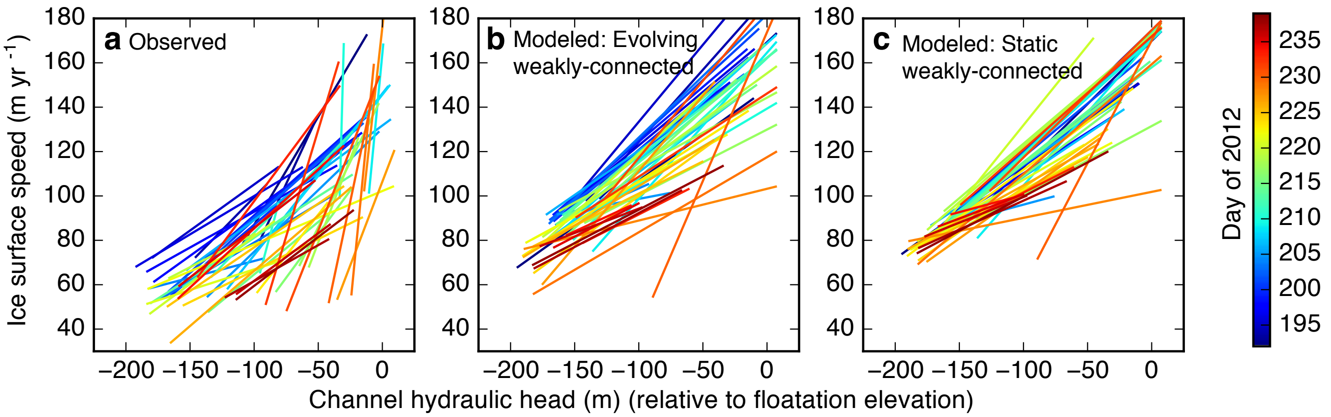

Supplementary Figure 6: **Seasonal evolution of the relationship between subglacial water pressure and ice speed with $f_w = 0.50$.** Each plot shows the minimum and maximum daily values of hydraulic head in the moulin-channel system and ice surface speed for the second half of the 2012 summer. **a** Observed relationship showing seasonal hysteresis of lowering ice speed for the same moulin head as summer progresses. Modified from Andrews et al.[2]. **b** Modeled relationship with increasing permeability of the weakly-connected regions of the bed. **c** Modeled relationship from control simulation with static weakly-connected system.

Supplementary Table 1: Physical constants and model parameters.

**Physical constants**		
ρ_w	density of water	1000 kg m^{-3}
ρ_i	density of ice	910 kg m^{-3}
g	gravitational acceleration	9.81 m s^{-2}
L	latent heat of fusion of water	3.35×10^5 J kg^{-1}
η_w	viscosity of water	1.7×10^{-3} Pa s
**Model numerics**		
$\Delta x = \Delta y$	horizontal grid spacing	200 m
Δt	time step	2 hr
**Subglacial hydrology model parameters**		
h_r	height of bedrock bumps	0.2 m
l_r	wavelength of bedrock bumps	1 m
G	geothermal heat flux	0.06 W m^{-2}
k_0	permeability constant for distributed system	3.5×10^{-6}
f_w	area fraction of weakly-connected system	0.67
A	flow law parameter of basal ice	6.4×10^{-24} s^{-1} Pa^{-3}
$\tau_{\mathbf{b}}$	basal traction	0.1 MPa
F	channel roughness	150 kg m$^{-8/3}$
r_w	stress transfer factor for weakly-connected cavities	0.13
Δs	radius of weakly-connected cavity patches	10 m
P_w	perimeter of weakly-connected cavity patches per grid cell	5355 m
$k_{0w_{winter}}$	base permeability constant for weakly-connected cavities	4.3×10^{-15}
k_{rate}	summer permeability change rate for weakly-connected cavities	1.2×10^{-12} yr^{-1}
**Ice dynamics model parameters**		
C	Coulomb friction coefficient	0.045
λ_{max}/m_{max}	ratio of controlling bedrock bump wavelength to maximum slope	0.01 m
τ_b°	Strength of basal "sticky" spots	55 kPa

Supplementary Table 2: Till model parameters.

Parameter	Description	Model value	Reference values and source
N_0	reference normal effective pressure	1000 Pa	1000 Pa; van der Wel, et al.[29]
e_0	void ratio at N_0	0.6	0.6; van der Wel, et al.[29]
C_c	coefficient of compressibility	0.15	0.25; van der Wel, et al.[29]
k_{t0}	reference permeability	4.5×10^{-14} m^2	2.9×10^{-13} m^2; Boulton, et al.[24]
m_t	permeability factor	4.7×10^{-6}	5.6×10^{-6}; Boulton, et al.[24]
$N_{t,max}$	maximum effective pressure in permeability relation	3.0×10^6 Pa	1.0×10^6 Pa; Boulton, et al.[24]
$N_{t,min}$	maximum effective pressure in permeability relation	3.0×10^5 Pa	1.0×10^5 Pa; Boulton, et al.[24]

Supplementary Table 3: Comparison of idealized model domain with field sites FOXX and GULL.

	Model	FOXX	GULL
Surface elevation (m)	745	707	880
Ice thickness(m)	745	620	700
Hydraulic head floatation elevation (m)	678	651	817
Surface slope (m/m)	0.015	0.029	0.023
Distance from terminus (km)	25	22	28

Supplementary Methods

The distributed and channelized drainage components used were previously described by Hoffman et al.[1]. Primary modifications from the previous description are changes to model nonlinear creep closure of subglacial cavities and the implementation of the new weakly-connected cavity system created to simulate the observations at drill site FOXX[2,3] as described in Methods. Complete descriptions of the distributed and channelized drainage components are provided here for clarity. A complete list of model parameters is provided in Supplementary Table 1.

Distributed drainage model. Distributed drainage is modeled as a two-dimensional macroporous sheet[4–6]. Conservation of mass of water is described by

$$\frac{\partial h}{\partial t} + \nabla \cdot \mathbf{q} = \frac{m}{\rho_w} - \frac{\gamma_c}{A_d} - \frac{\gamma_w}{A_d}\,, \tag{1}$$

where h is the water thickness in an equivalent macroporous sheet (m), $\mathbf{q}$ is the areal discharge of the sheet (m^2 s^{-1}), m is the melt rate (kg m^{-2} s^{-1}), ρ_w is the density of water (kg m^{-3}), γ_c is water transferred between the distributed system and channel (m^3 s^{-1}), and γ_w is water transferred between the distributed system and the weakly-connected system (m^3 s^{-1}). A_d is the area of the distributed system within each grid cell (m^2), defined as $(1 - f_w)\Delta x \Delta y$.

The evolution of subglacial cavity space within the sheet is described by a balance between opening of cavities by sliding and creep closure[7] of the ice above following a Glen flow rheology[8]:

$$\frac{\partial h}{\partial t} = |\mathbf{u_b}|\frac{h_r - h}{l_r} - \frac{2A}{27} h N_d^3\,, \tag{2}$$

where $\mathbf{u_b}$ is the sliding velocity, A is the temperature dependent rate factor, N_d is ice effective pressure in the distributed system, and h_r and l_r are parameters describing the height and wavelength, respectively, of bumps on the bed.

The distributed system assumes a Darcy style[9,10] flow law[6],

$$\mathbf{q} = -\frac{k_0 h^3}{\eta_w}\sqrt{1 - f_w}\nabla\phi_d\,, \tag{3}$$

where η_w is the viscosity of water (Pa s) and ϕ_d is the hydraulic potential (Pa) in the distributed system. From a Darcy flow perspective, the permeability (m^2) is $k_0 h^2$ where k_0 is a permeability

constant and the power to which h is raised is chosen as an analog for laminar flow assumed to occur within the distributed system[6]. The term $\sqrt{1-f_w}$ is a correction that accounts for the fact that flow through the distributed system only occurs within some fraction of the grid cell (as discussed further below).

Energy for local melting comes from the geothermal flux, G (W m^{-2}) and frictional heating from ice sliding. Assuming isothermal ice conditions at the bed, this energy is given by

$$mL = G - \mathbf{u_b} \cdot \tau_\mathbf{b} \,, \tag{4}$$

where $\tau_\mathbf{b}$ is the basal traction vector and L is the latent heat of fusion of water.

Channelized drainage model. Channelized drainage[7,11] uses a similar set of equations, though the physical processes which dominate system evolution differ. A single channel is imposed along the center of the domain (Fig. 2b). While the channel area is able to freely evolve, it is assumed that the map-plane area of the channel remains small relative to that covered by the distributed and weakly-connected systems. Therefore, it is implemented as a linear feature in the computational domain and takes up no area on the two-dimensional model grid (Fig. 2b).

The mass balance of water within the linear channel is expressed by

$$\frac{\partial S}{\partial t} + \frac{\partial Q}{\partial x} = \frac{M}{\rho_w} + \omega + \omega_s + \frac{\gamma_c}{\Delta x} \,, \tag{5}$$

where S is channel area (m^2), Q is volumetric channel flowrate (m^3 s^{-1}), M is melt rate within the channel (kg m^{-1} s^{-1}), ω is an englacial source term accounting for surface meltwater draining to the bed (m^2 s^{-1}), and ω_s is an englacial source term accounting for release of water stored in a near-surface englacial reservoir (m^2 s^{-1}).

Observations indicate that moulin water pressure does not significantly exceed floatation pressure (Fig. 4b); therefore we include a parameterization of a near-surface englacial storage system (represented by ω_s). We assume this reservoir is composed of crevasses and fractures[12] into which water can back up when water levels in the moulin are high. Storage in the moulin itself is also possible, but the observations suggest this missing storage process is important only when moulin water levels are near the ice surface, making crevasses a more likely candidate. Subglacial storage due to hydraulic jacking is another possibility[13–16] that we do not consider due to its substantial complexity[15,16]. To reproduce the observed behavior, we add a simple parameterization

of crevasse storage and release to the model. If meltwater input causes channel hydraulic head to exceed 60 m below the ice surface in any grid cell, ω is decreased within that grid cell until channel hydraulic head matches the elevation corresponding to 60 m below the ice surface, with this value chosen as a typical depth of the brittle crevasse zone[12,17]. To conserve mass, the excess melt input is stored in a near-surface englacial reservoir that releases a steady fraction of its stored water volume over time ω_s, following

$$\omega_s = \frac{V_s}{t_s}\,, \tag{6}$$

where V_s is the volume of water currently stored and t_s is a time constant controlling the rate of release. This is a simplified version of the englacial hydrologic system described by Flowers and Clarke[18]. The time constant determining the constant for water release is tuned to $t_s = 2$ days to best reproduce the moulin head variations following the large melt event that occurs around day 230.

The evolution of channel area is given by the balance of melt opening and creep closure[7] following a Glen flow rheology[8]:

$$\frac{\partial S}{\partial t} = \frac{M}{\rho_i} - \frac{2A}{27} S N_c^3\,, \tag{7}$$

where N_c is the effective pressure in the channel.

Melt within the channel is entirely from dissipation:

$$ML = Q\frac{\partial \phi_c}{\partial x}\,, \tag{8}$$

where ϕ_c is the hydraulic potential in the channel.

Within the channel, we use Manning's law for turbulent flow,

$$FQ^2 = S^{8/3}\frac{\partial \phi_c}{\partial x}\,, \tag{9}$$

where F is a roughness parameter (kg m$^{-8/3}$).

The channel is coupled to the distributed system model on either side by calculating a flux, γ_c, between the surrounding distributed system and the channel based on the Darcy flow law (Supplementary Equation (3)):

$$\gamma_c = -\frac{k_0 h^3}{\eta_w}\frac{\phi_c - \phi_d}{\frac{1}{2}\Delta y}\Delta x\,. \tag{10}$$

Note that exchange between the channel and the distributed drainage component can be in either direction, with water always moving from the component with higher hydraulic potential to that with lower. There is no exchange between the channel and weakly-connected drainage, but indirect interactions between the channel and weakly-connected drainage occur via the intermediary distributed system.

Subglacial till model. Both modeling[19,20] and observations[21,22] have argued for the existence of significant amounts of till underlying marginal regions of the GrIS. The rough topography of our study area makes it unclear to what extent till coverage is continuous. Additionally, the presence of till does not exclude the formation of subglacial hydrologic features typically associated with hard beds, including channels[23] and lee-side cavities[24]. Furthermore, canals eroded into till may behave similarly to cavity-based distributed systems[6,23,25,26]. Thus, our models for channelized, distributed, and weakly-connected drainage may all be applicable in the presence of till. Here we address the possibility that the weakly-connected system is composed entirely of till without significant cavitation. Based on a simple till model formulation, we find that a weakly-connected system composed of till could have a similar hydrologic signature to our proposed weakly-connected cavity system.

The alternative formulation for the weakly-connected system is derived from the assumption that the weakly-connected regions are composed of heterogeneously distributed subglacial till. As for the weakly-connected cavity model, we keep the formulation as simple as possible while including the basic processes expected to occur. As such, this simple model ignores many of the complicated behaviors of till such as dilation, comminuation, and piping[27]. Similar to the weakly-connected cavity model, we conceptualize the till model as patchy till with low permeability surrounded by the distributed system (as in Fig. 2b Inset). We assume the same characteristic spatial dimensions, P_w and A_w, for the till model as for the weakly-connected cavity model.

Till porosity, ψ, is defined as the ratio of fluid volume (i.e., water), V_f to solids volume, V:

$$\psi = \frac{V_f}{V} \,. \tag{11}$$

Void ratio, e_t, is a function of porosity:

$$e_t = \frac{\psi}{1 - \psi} \,. \tag{12}$$

Effective normal stress in the till, N_t, can be written in terms of void ratio[27–29]:

$$N_t = N_0 10^{\frac{e_0 - e_t}{C_c}} , \tag{13}$$

where N_0 is the reference normal effective pressure (Pa), e_0 is the void ratio at N_0, and C_c is the coeffecient of compressibility.

Exchange of water from the till to the surrounding distributed system, γ_t, follows Darcy flow through a porous medium[17,27] similar to Methods Equation (6), and takes the place of γ_w in Supplementary Equation (1):

$$\gamma_t = -\frac{k_t h_t}{\eta_w} \frac{\phi_t - \phi_d}{\Delta s} P_w , \tag{14}$$

where h_t is calculated as V_f per unit area.

For the till model we can make use of an empirical relationship between effective pressure and permeability[24,27],

$$k_t = k_{t0} \exp(-m_t N_t) , \tag{15}$$

where k_{t0} and m_t are material properties of the till. Boulton, et al.[24] describe the range of effective pressure over which this empirical relation is expected to hold as $\sim 10^5$–10^6 Pa, and the pressure dependence of permeability would be expected to be much weaker outside of this range. In tuning our model to match borehole observations, we consider these limits as tunable parameters ($N_{t,max}$ and $N_{t,min}$), but keep them the same order of magnitude. In our model, permeability then remains at its limiting values outside of this range. For our application we consider N_t in Supplementary Equation (15) to be the maximum of the effective pressure in the distributed system and the till. In other words, water flow out of the till is limited by the region of the till with the higher effective pressure, and therefore, the smaller permeability – either the interface of the till with the surrounding drainage system forms a low permeability “plug” when the distributed system has a larger effective pressure or the interior of the till is unable to transmit water fast enough for the interface region to accept it when the till itself is at higher effective pressure.

We find that in order to reproduce diurnal variations in water pressure, the till model requires an explicit representation of diurnal variations in normal stress. We introduce a parameterization for this by making the ice overburden pressure experienced by the till, p_{ice_t}, a function of the effective pressure in the distributed system in the same grid cell:

$$p_{ice_t} = \rho_i g H + r_t N_d . \tag{16}$$

The adjustment factor, r_t, represents the ratio of mechanical support between the two systems, which in theory would be a function of the relative area of the two systems as well as the fraction of mechanical support provided by bedrock and clasts[30]. We tune r_t to 0.08 as a good fit to both the winter pressure in boreholes and the amplitude of diurnal pressure variations observed in the boreholes during summer.

We apply this simple till model by tuning available parameters to yield hydraulic head in the till similar to that observed in boreholes. Tuned parameter values are generally within the same order of magnitude as reference values (Supplementary Table 2). Water pressure in the till calculated by the till model is qualitatively similar to the water pressure calculated by the weakly-connected cavity model (Supplementary Fig. 4).

Note that while we do not perform ice dynamics calculations using results from the till model, we would expect similar qualitative results as the weakly-connected cavity model, based on the similarity of the basal traction relation for till to that for hard beds[17]. Till yield strength, τ_f, follows the Mohr-Coulomb rule[17,27–29,31],

$$\tau_f = c_t + Ntan(\phi_f) \approx Ntan(\phi_f)\,, \tag{17}$$

where c_t is the apparent cohesion of till, which is typically small, and ϕ_f is the angle of internal friction. To represent regions of hard and soft bed over scales relevant for ice dynamics, area weighted fractions of basal traction from the two systems as in Methods Equation (13) are appropriate[17]. Thus, τ_{bw} in Methods Equation (13) would be defined by τ_f in Supplementary Equation (17). Note that in both the weakly-connected cavity model and the till model, the basal traction contribution of the weakly-connected system is equal to the effective pressure in the weakly-connected system times a friction factor (c.f., Supplementary Equation (17) and Methods Equation (11)). Therefore, for appropriately chosen parameter values, similar values of basal traction, and therefore velocity, will be generated from both models.

Supplementary References

1. Hoffman, M. & Price, S. Feedbacks between coupled subglacial hydrology and glacier dynamics. *Journal of Geophysical Research: Earth Surface* **119**, 1–23 (2014).

2. Andrews, L. C. *et al.* Direct observations of evolving subglacial drainage beneath the Greenland Ice Sheet. *Nature* **514**, 80–83 (2014). URL *http://www.nature.com/doifinder/10.1038/nature13796*.

3. Ryser, C. *et al.* Caterpillar-like ice motion in the ablation zone of the Greenland ice sheet. *Journal of Geophysical Research Earth Surface* **119**, 1–14 (2014).

4. Flowers, G. E. & Clarke, G. K. A multicomponent coupled model of glacier hydrology 1. Theory and synthetic examples. *Journal of Geophysical Research* **107**, 2287 (2002). URL *http://doi.wiley.com/10.1029/2001JB001122*.

5. Schoof, C. Ice-sheet acceleration driven by melt supply variability. *Nature* **468**, 803–806 (2010). URL *http://www.nature.com/nature/journal/v468/n7325/full/nature09618.html*.

6. Hewitt, I. J. Modelling distributed and channelized subglacial drainage: the spacing of channels. *Journal of Glaciology* **57**, 302–314 (2011). URL *http://openurl.ingenta.com/content/xref?genre=article&issn=0022-1430&volume=57&issue=202&spage=302*.

7. Nye, J. F. Water flow in glaciers: jökulhlaups, tunnels and veins. *Journal of Glaciology* **17**, 181–207 (1976).

8. Glen, J. W. The Creep of Polycrystalline Ice. *Proceedings of the Royal Society A: Mathematical, Physical and Engineering Sciences* **228**, 519–538 (1955). URL *http://rspa.royalsocietypublishing.org/cgi/doi/10.1098/rspa.1955.0066*.

9. Darcy, H. *Les Fontaines Publiques de la Ville de Dijon* (Victor Dalmont, Paris, 1856).

10. Freeze, R. & Cherry, J. *Groundwater* (Prentice-Hall, Englewood Cliffs, NJ, 1979).

11. Röthlisberger, H. Water pressure in intra- and subglacial channels. *Journal of Glaciology* **11**, 177–203 (1972).

12. McGrath, D., Colgan, W., Steffen, K., Lauffenburger, P. & Balog, J. Assessing the summer water budget of a moulin basin in the Sermeq Avannarleq ablation region, Greenland ice sheet. *Journal of Glaciology* **57**, 954–964 (2011). URL *http://openurl.ingenta.com/content/xref?genre=article&issn=0022-1430&volume=57&issue=205&spage=954*.

13. Das, S. B. *et al.* Fracture Propagation to the Base of the Greenland Ice Sheet During Supraglacial Lake Drainage. *Science* **320**, 778–781 (2008).

14. Pimentel, S. & Flowers, G. E. A numerical study of hydrologically driven glacier dynamics and subglacial flooding. *Proceedings of the Royal Society A: Mathematical, Physical and Engineering Sciences* **467**, 537–558 (2010). URL *http://rspa.royalsocietypublishing.org/cgi/doi/10.1098/rspa.2010.0211*.

15. Tsai, V. C. & Rice, J. R. A model for turbulent hydraulic fracture and application to crack propagation at glacier beds. *Journal of Geophysical Research* **115** (2010). URL *http://www.agu.org/pubs/crossref/2010/2009JF001474.shtml*.

16. Dow, C. F. *et al.* Modeling of subglacial hydrological development following rapid supraglacial lake drainage. *Journal of Geophysical Research: Earth Surface* **120**, 1127–1147 (2015).

17. Cuffey, K. & Paterson. *The Physics of Glaciers* (Butterworth-Heinneman, Amsterdam, 2010), 4th edn.

18. Flowers, G. E. A multicomponent coupled model of glacier hydrology 2. Application to Trapridge Glacier, Yukon, Canada. *Journal of Geophysical Research* **107**, 2288 (2002). URL *http://doi.wiley.com/10.1029/2001JB001124*.

19. Bougamont, M. *et al.* Sensitive response of the Greenland Ice Sheet to surface melt drainage over a soft bed. *Nature Communications* **5**, 5052 (2014). URL *http://www.nature.com/doifinder/10.1038/ncomms6052*.

20. Shapero, D. R., Joughin, I. R., Poinar, K., Morlighem, M. & Gillet-chaulet, F. Basal resistance for three of the largest Greenland outlet glaciers. *Journal of Geophysical Research: Earth Surface* **121**, 168–180 (2016).

21. Dow, C. *et al.* Seismic evidence of mechanically weak sediments underlying Russell Glacier, West Greenland. *Annals of Glaciology* **54**, 135–141 (2013).

22. Walter, F., Chaput, J. & Luthi, M. P. Thick sediments beneath Greenland's ablation zone and their potential role in future ice sheet dynamics. *Geology* **42**, 487–490 (2014). URL *http://geology.gsapubs.org/cgi/doi/10.1130/G35492.1*.

23. Walder, J. & Fowler, A. Channelized subglacial drainage over a deformable bed. *Journal of Glaciology* **40**, 3–15 (1994).

24. Boulton, G. S., Dent, D. L. & Morris, E. M. Subglacial shearing and crushing, and the role of water pressures in tills from South-East Iceland. *Geografiska Annaler Series A: Physical Geography* **56**, 135–145 (1974).

25. Ng, F. S. L. Canals under sediment-based ice-sheets. *Annals of Glaciology* **30**, 146–152 (2000).

26. Catania, G. & Paola, C. Braiding under glass. *Geology* **29**, 259–262 (2001).

27. Clarke, G. K. C. Subglacial till: A physical framework for its properties and processes. *Journal of Geophysical Research* **92**, 9023–9036 (1987).

28. Tulaczyk, S., Kamb, W. B. & Engelhardt, H. F. Basal mechanics of Ice Stream B, West Antarctica 2. Undrained plastic bed model. *Journal of Geophysical Research* **105**, 483–494 (2000).

29. van der Wel, N., Christoffersen, P. & Bougamont, M. The influence of subglacial hydrology on the flow of Kamb Ice Stream, West Antarctica. *Journal of Geophysical Research: Earth Surface* **118**, 97–110 (2013). URL *http://doi.wiley.com/10.1029/2012JF002570*.

30. Murray, T. & Clarke, G. K. C. Black-box modeling of the subglacial water system. *Journal of Geophysical Research* **100**, 10231–10245 (1995).

31. Iverson, N. R., Hooyer, T. S. & Baker, R. V. Ring-shear studies of till deformation: Coulomb-plastic behavior and distributed strain in glacier beds. *Journal of Glaciology* **44**, 634–642 (1998).
